# FTO promotes cancer progression by regulating VGLL4 m6A levels to activate STAT3 signaling in triple-negative breast cancer

**DOI:** 10.1016/j.jbc.2026.113242

**Published:** 2026-06-09

**Authors:** Hongming Song, Jihong Lu, Yining Zhang, Zhidong Lv, Yuhua Song, Wenqin Wang, Yaoyu Xu, Tiantian Bian, Yongmei Wang, Lei Zhang, Xiaohui Su, Dengfeng Li, Zhaohe Niu

**Affiliations:** 1 Breast Disease Center, The Affiliated Hospital of Qingdao University, Qingdao, Shandong, China; 2 Department of Breast Surgery, The First Hospital of China Medical University, Shenyang, Liaoning, China; 3 School of Health and Life Sciences, University of Health and Rehabilitation Sciences, Qingdao, Shandong, China; 4 College of Chemistry and Chemical Engineering, Anyang Normal University, Anyang, Henan, China; 5 Department of Breast and Thyroid Surgery, Shanghai Tenth People's Hospital, Tongji University School of Medicine, Shanghai, China

**Keywords:** triple-negative breast cancer, FTO, m6A modification, cancer progression, VGLL4-STAT3 signaling

## Abstract

Triple-negative breast cancer (TNBC) is a highly aggressive malignancy with poor prognosis and high mortality rates. Recent studies have highlighted the critical role of N6-methyladenosine (m6A) modification in cancer progression, yet the specific function of the m6A regulatory factor FTO in TNBC remains unclear. In this study, the expression of FTO in clinical samples was obtained from the GEO dataset, and correlation analysis between FTO expression and patient clinicopathological parameters was conducted. Functional assays, including MTT, colony formation, wound healing, transwell experiments, and *in vivo* tumor formation assay, were conducted to investigate the effect of FTO on the cellular behaviors of TNBC. qRT-PCR, immunohistochemistry (IHC), and Western blotting (WB) were conducted to examine the expression levels of FTO in TNBC, transcriptome sequencing, M6A-RNA immunoprecipitation (MeRIP), RNA immunoprecipitation (RIP), and RNA stability assays were employed to investigate the underlying mechanisms regulated by FTO. We confirmed that FTO is highly expressed in TNBC tissues. Furthermore, high FTO expression was associated with worse patient prognosis in TNBC. Functional assays demonstrated that FTO overexpression promotes TNBC cell proliferation, migration. *In vivo* studies demonstrated that overexpression of FTO promotes tumor growth in TNBC cells. Mechanistically, VGLL4 was identified as the target gene for FTO-mediated m6A modifcation. Our findings reveal that FTO drives TNBC progression by modifying the m6A level of VGLL4, leading to the activation of the STAT3 signaling pathway. In summary, these findings reveal the role of FTO-mediated m6A demethylation in TNBC and indicate potential therapeutic strategies for this aggressive disease.

Breast cancer (BC) is the most commonly diagnosed cancer among women worldwide (1, 2). Triple-negative breast cancer (TNBC), a particularly aggressive BC subtype, is defined by the absence of estrogen receptor (ER), progesterone receptor (PR), and human epidermal growth factor receptor 2 (HER2) expression. As a result, TNBC does not respond to endocrine therapies or HER2-targeted treatments (3), leading to higher recurrence rates, increased metastatic potential, and worse clinical outcomes compared to other BC subtypes. However, a poor understanding of the pathogenesis of TNBC at the molecular level has restricted advances in its treatment approaches.

N6-methyladenosine (m6A) methylation is one of the post-transcriptional RNA modifications. m6A is a methylation modification of the sixth N position of adenine (A) catalyzed by methyltransferase, and is most abundant in messenger RNAs (mRNAs) and noncoding RNAs (4). The m6A modification is dynamically reversible. It is catalyzed by m6A methyltransferase (METTL3, METTL14, and WTAP), also known as "the writer". m6A demethylases (FTO and ALKBH5) are also known as "erasers" that can directly remove m6A modifications from mRNA. In addition, specific RNA-binding proteins, also known as "readers," such as YTH domain family proteins 1 to 3 (YTHDF1–3) (5, 6), and insulin-like growth factor 2 mRNA-binding proteins 1 to 3 (IGF2BP1–3) (7) are responsible for specifically recognizing and binding methylation sites to perform specific biological functions (8). Accumulating studies indicate that m6A-mediated regulation of RNA biological processes (processing, splicing, nucleation, translation, and stability), whose dysregulation plays pivotal roles in the pathogenesis of human disorders, including cancers (9). However, the significance of m6A modification and the underlying regulatory mechanism in TNBC has not been fully elucidated.

The fat mass and obesity-associated protein (FTO) regulates metabolic processes related to food utilization and energy consumption, and it exhibits a strong correlation with body mass index and obesity in humans. Moreover, it also affects the development of adipose tissue (10, 11). FTO is frequently upregulated in cancers and functions as a tumor promoter, it drives tumor progression by enhancing cancer cell proliferation, maintaining cancer stem cell self-renewal capacity, and remodeling the tumor immune and metabolic microenvironment through m6A demethylation of target mRNAs, thereby modulating their stability (12). Furthermore, oncogenic metabolic pathways activated by FTO have been shown to promote tumorigenesis and chemoresistance in cancer cells (13, 14, 15). Although FTO has been demonstrated to play critical roles in various cancers, the specific mechanism of FTO in tumorigenesis and chemoresistance of TNBC remains largely unclear. Therefore, this study aimed to analyze FTO expression in TNBC samples and investigate its functional impact on TNBC cells to further elucidate the regulatory mechanisms of FTO in TNBC.

In this study, we explored the role of demethylase FTO and its relationship with clinicopathological features in TNBC. High FTO expression was associated with worse patient prognosis in TNBC. We found that FTO plays a key role in promoting the proliferation and migration of TNBC cells. Mechanistically, we demonstrated that FTO drives oncogenesis by regulating the VGLL4/STAT3 signaling pathway. Overall, our results provide new insight into the potential mechanism of FTO-mediated m6A modification in modulating the progression of TNBC.

## Results

### Characterization of FTO in TNBC tissues

**Figure 1:**
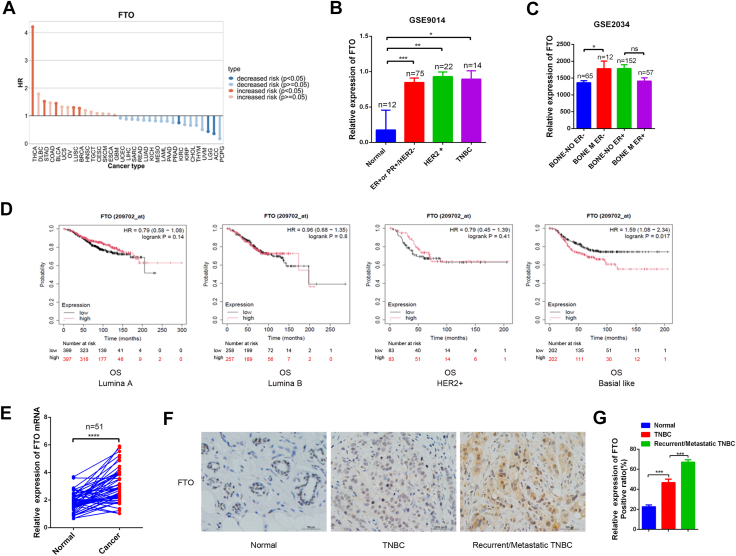
**The expression profile of FTO in TNBC tissues.***A*, HR for FTO expression across 33 cancer types. *B*, FTO expression across breast cancer molecular subtypes (ER+ or PR+/Her2-, Her2+, and TNBC). *C*, the expression characteristics of FTO in breast cancer related to ER status and bone metastasis. *D*, overall survival analysis of FTO expression across molecular subtypes of breast cancer. *E*, relative expression of FTO in TNBC tissues and adjacent normal tissues. *F–G*, IHC analysis of FTO in normal adjacent, primary TNBC, and recurrent/metastatic tissues. *∗p < 0.05, ∗∗p < 0.01, ∗∗∗p < 0.001, ∗∗∗∗p < 0.0001*. NS, not significant.

According to the TCGA survival analysis from the TISCH Database (http://tisch.comp-genomics.org), the Cox proportional hazards model was applied to evaluate the FTO gene across 33 cancer types, yielding hazard ratios (HR) and corresponding *p*-values. Specifically, the HR for FTO expression was found to be greater than 1 in breast cancer (*p* < 0.05), suggesting that elevated FTO expression is associated with an increased risk of mortality and disease progression (Fig. 1*A*). Notably, we found that the core m6A demethylase FTO was significantly upregulated across breast cancer molecular subtypes (Her2+, ER+ or PR+/Her2-, and TNBC) compared to normal breast tissues, based on our analysis of 111 breast tumors and 12 non-tumorous normal breast samples (GSE9014, Fig. 1*B*). We further confirmed FTO was significantly up-regulated in ER-breast tumors with bone metastasis compared with ER-breast tumors without bone metastasis. However, in ER+ breast tumors, there was no significant difference in FTO expression between those with bone metastasis and those without. (GSE2034, Fig. 1*C*). Through an online database of Kaplan-Meier Plotter survival analysis (https://www.kmplot.com/analysis/), we assessed the association between FTO expression and overall survival (OS) across various molecular subtypes of breast cancer. The results indicated no significant difference in OS between patients with high and low FTO expressions within Luminal A, Luminal B, and HER2-enriched subgroups. However, in the basal-like subgroup, high FTO expression was significantly associated with reduced OS compared to the low-expression group, suggesting that elevated FTO expression may correlate with a poorer prognosis in TNBC (Fig. 1*D*). We next evaluated FTO expression in 51 randomly selected TNBC tissues along with matched adjacent normal tissues and observed a marked upregulation of FTO in TNBC samples compared to normal counterparts (Fig. 1*E*). Similarly, the results showed that FTO expression was highest in TNBC cells (BT-549, HCC1937, MDA-MB-468), followed by luminal cells (T47D) and HER2-positive cells (SK-BR-3), while luminal cells (MCF-7) and TNBC cells (MDA-MB-231) exhibited relatively lower FTO levels (Fig. S1*A*). We further assessed the relationship between FTO levels and clinicopathological characteristics among the 51 patients. Our analysis revealed a positive correlation of FTO expression with lymph node metastasis, Ki-67 index, and TNM tumor stage (Table 1). Consistent with these findings, IHC analysis showed that FTO expression was notably elevated in TNBC tissues, as well as in recurrent or metastatic tissues, relative to adjacent normal tissues (Fig. 1, *F* and *G*).

**Table 1:** Association between FTO expression and clinicopathological characteristics in TNBC (n = 51)

Characteristics	No. of cases	FTO expression		*p* value
		≥1.655	<1.655	
Age (year)				0.3314
≥50	28	16	12	
<50	23	10	13	
Tumor size (mm)				0.1656
≥20	34	15	19	
<20	17	11	6	
Ki-67 index				0.0327
≥30	32	20	12	
<30	19	6	13	
Lymph node metastasis				0.0203
No	35	14	21	
Yes	16	12	4	
Tumor recurrence or metastasis				0.244
No	44	21	23	
Yes	7	5	2	
TNM tumor stage				
I + II	30	11	19	0.0145
III + IV	21	15	6	

### FTO-exerted oncogenic role in TNBC cells

**Figure 2:**
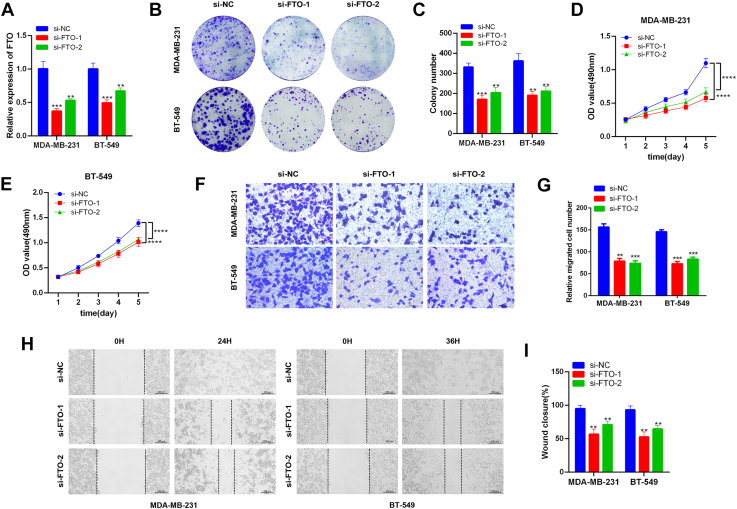
**Silencing FTO suppresses the proliferation and migration of TNBC cells.***A*, expression of FTO was confirmed by RT-qPCR in MDA-MB-231 and BT-549 cell lines transfected with si-NC or si-FTO. *B–C*, colony formation ability of TNBC cells after FTO knockdown. *D–E*, MTT assay assessing proliferation after FTO silencing in MDA-MB-231 and BT-549 cells. *F–G*, transwell assay evaluating migration after FTO knockdown in TNBC cells. *H-I*, wound healing assay evaluating migration after FTO knockdown in TNBC cells. *∗∗p < 0.01, ∗∗∗p < 0.001, ∗∗∗∗p < 0.0001.*

**Figure 3:**
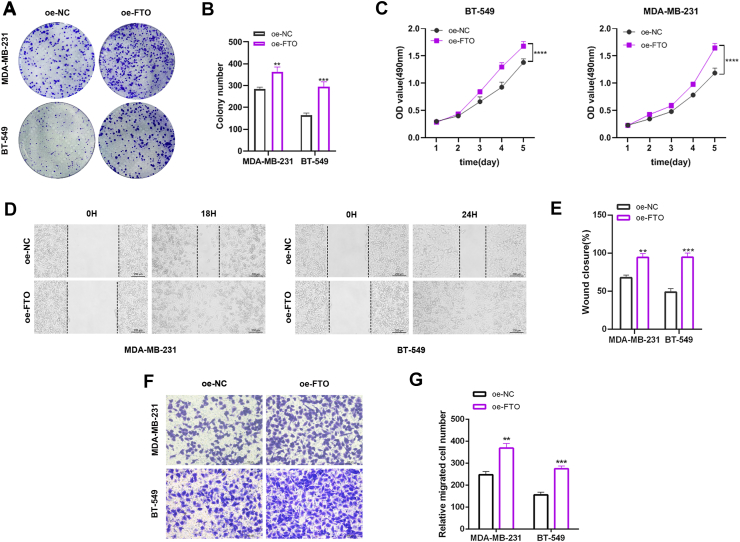
**Overexpression of FTO promotes the proliferation and migration of TNBC cells.***A–B*, colony formation assay evaluating proliferative capacity after FTO overexpression in TNBC cells. *C*, MTT assay assessing proliferation after FTO overexpression in TNBC cells. *D–E*, wound healing assay analyzing cell migration following FTO overexpression in TNBC cells. *F–G*, transwell assay evaluating cell migration following FTO overexpression in TNBC cells. *∗∗p < 0.01, ∗∗∗p < 0.001, ∗∗∗∗p < 0.0001.*

To investigate the biological function of FTO in TNBC, we transfected MDA-MB-231 and BT-549 cells with two specific siRNAs against FTO (si-FTO-1 and si-FTO-2) (Fig. 2*A*). Both MTT and colony formation assays revealed that FTO knockdown significantly inhibited the proliferation of these two cell lines (Fig. 2, *B*–*E*). Furthermore, transwell and wound healing assays demonstrated that FTO depletion impaired cell migration (Fig. 2, *F*–*I*). We next generated an FTO-overexpressing plasmid. Stable expression of this construct significantly elevated FTO levels compared to the empty vector, which served as negative control (NC). Ectopic FTO expression markedly promoted both proliferation (Fig. 3, *A*–*C*) and migration (Fig. 3, *D*–*G*) in MDA-MB-231 and BT-549 cells. Collectively, these results indicate that FTO plays a critical role in promoting the proliferation and migration of TNBC cells *in vitro*.

### FTO promotes tumor growth of TNBC *in vivo*

**Figure 4:**
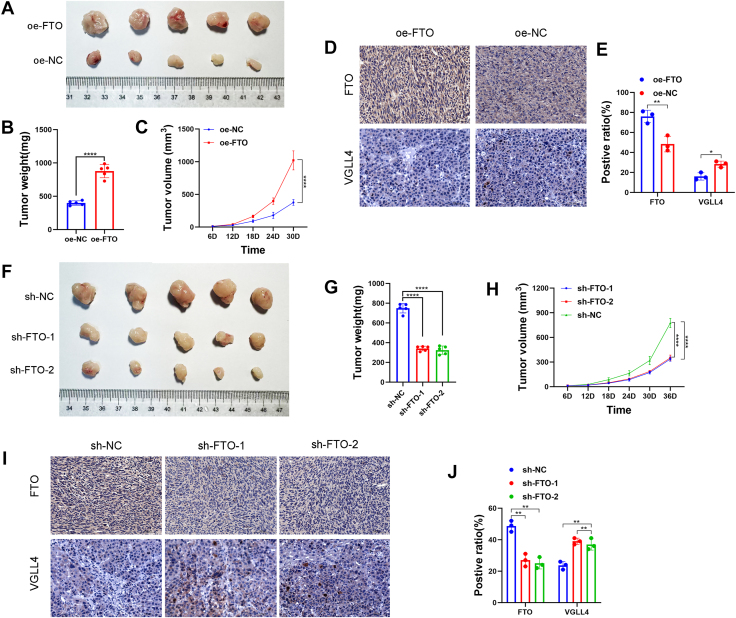
**Overexpression of FTO promotes TNBC tumor growth *in vivo*.***A–C*, Gain-of-function studies in orthotopic TNBC xenograft models. FTO overexpression markedly promotes tumor growth and results in larger dissected tumors (*A*), increased average tumor weight (*B*), and accelerated tumor volume growth (*C*) compared to oe-NC controls. *D–E*,Immunohistochemistry (IHC) staining analysis of tumor sections (*D*) shows elevated FTO protein abundance and diminished VGLL4 staining upon FTO overexpression(*E*). *H*, Loss-of-function studies in orthotopic TNBC xenograft models. FTO knockdown markedly reduces tumor growth and results in smaller dissected tumors (*F*), decreased tumor weight (*G*), and slower tumor volume growth (*H*) compared to shNC controls. *I–J*, IHC analysis of orthotopic tumor sections (*I*) shows reduced FTO and increased VGLL4 staining upon FTO knockdown (*J*), indicating reduced proliferative capacity *in vivo*. *∗p < 0.05, ∗∗p < 0.01, ∗∗∗p < 0.001, ∗∗∗∗p < 0.0001.*

To further investigate the role of FTO in TNBC *in vivo*, we established xenograft models using MDA-MB-231 cells with stable FTO overexpression or knockdown. These cells or their corresponding control cells were orthotopically injected into the second mammary fat pads of nude mice (n = 5 per group). After approximately 5 weeks, the mice were euthanized, and the resulting tumors were excised and photographed (Fig. 4, *A*–*C*). Analysis of both tumor volume and weight demonstrated that FTO overexpression markedly promoted tumor growth, IHC staining on the tumor sections showed that overexpression of FTO significantly increased its own expression level compared to the NC group. Conversely, VGLL4 expression was markedly reduced in the FTO-overexpressing group (Fig. 4, *D* and *E*), indicating that FTO overexpression may negatively regulate VGLL4 expression. However, FTO knockdown significantly suppressed tumor progression in TNBC xenografts (Fig. 4, *F*–*H*). IHC analysis of tumor sections showed that FTO knockdown reduced FTO expression but upregulated VGLL4 expression compared to the sh-NC group (Fig. 4, *I* and *J*), indicating that FTO loss enhances VGLL4 expression.

### VGLL4 as a downstream target of FTO-mediated m6A modification

To identify the downstream effectors mediating FTO’s oncogenic function, we analyzed RNA-seq data (or m6A-seq data) of FTO-overexpressing cells. Among the differentially expressed genes with altered m6A peaks, we focused on VGLL4 because: (i) its mRNA contained a conserved m6A consensus motif (Fig. S1*B*); (ii) its expression was significantly downregulated upon FTO overexpression (Fig. S1, *C* and *D*); and (iii) VGLL4 is known as a tumor suppressor in the TNBC, consistent with FTO’s pro-tumor role.

**Figure 5:**
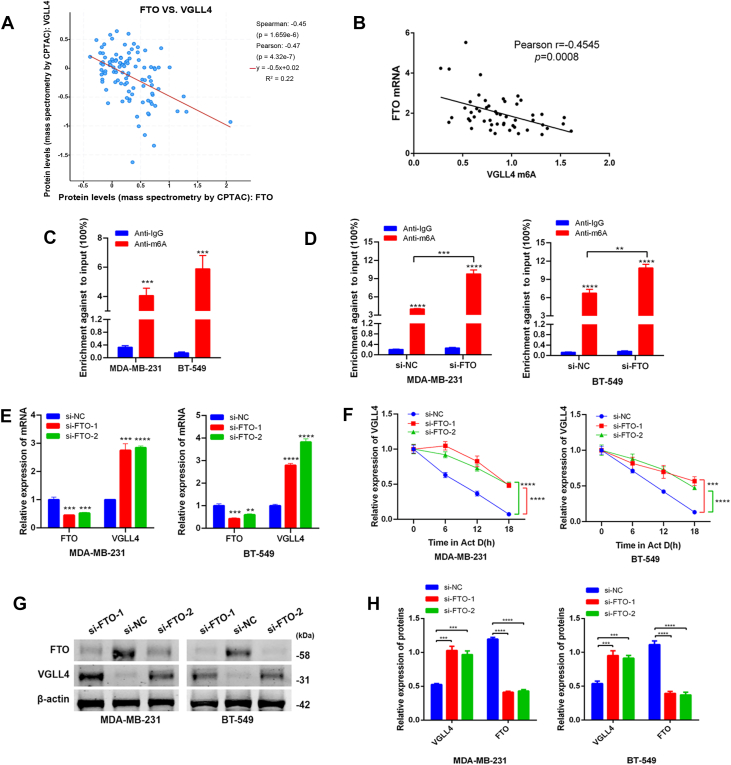
**FTO knockdown positively regulates VGLL4 expression in TNBC cells.***A*, Pearson’s correlation analysis revealed a negative correlation between FTO and VGLL4 expression in BC tissues. *B*, RT-qPCR confirmed the negative correlation between FTO and VGLL4 m6A abundance in TNBC tissues. *C*, the enrichment of VGLL4 m6A was detected after immunoprecipitation with anti-m6A in RIP assay, with anti-IgG as negative controls. *D*, the enrichment of VGLL4 m6A levels were evaluated in MDA-MB-231 and BT-549 cell lines transfected with si-FTO. *E*, the mRNA levels of VGLL4 and FTO were evaluated in MDA-MB-231 and BT-549 cell lines transfected with si-FTO. *F*, Analysis of VGLL4 mRNA stability by RT-qPCR after Actinomycin D treatment in FTO-knockdown MDA-MB-231 and BT-549 cells. *G–H*, the protein levels of VGLL4 and FTO were evaluated after FTO knockdown in MDA-MB-231 and BT-549 cells. *∗∗p < 0.01, ∗∗∗p < 0.001, ∗∗∗∗p < 0.0001.*

We employed the cBioPortal database (http://www.cbioportal.org/) to analyze genetic correlations of the FTO gene. Our analysis identified a prominent negative correlation between FTO and VGLL4 protein expression (Fig. 5*A*). Interrogation of SRAMP databases (http://www.cuilab.cn/sramp/) further showed that VGLL4 is enriched in m6A modification sites (Fig. S1*B*). Based on this finding, we analyzed the high-throughput sequencing expression profiles of the m6A demethylase FTO using datasets GSE129842 and GSE130349 (from the GEO database). Analysis of the GSE129842 dataset revealed a marked increase in VGLL4 m6A modification levels in FTO-knockout (KO) groups compared to wild-type (WT) controls (Fig. S1*C*). Conversely, findings from the GSE130349 dataset demonstrated a significant decrease in VGLL4 m6A modification abundance following FTO overexpression (OE) relative to WT groups (Fig. S1*D*). These findings indicate that FTO may regulate VGLL4 expression *via* an m6A-dependent mechanism. Furthermore, a significant negative correlation between FTO mRNA expression and VGLL4 m6A modification levels was observed in TNBC samples (Fig. 5*B*). We detected the m6A modification level of VGLL4 in MDA-MB-231 and BT-549 cells and observed that its enrichment was significantly higher in the m6A-IP samples than in the IgG-IP controls in both cell lines (Fig. 5*C*). In MDA-MB-231 and BT-549 cells, FTO knockdown significantly increased the m6A modification of VGLL4 compared with the control group (Fig. 5*D*). RT-qPCR analysis revealed that FTO knockdown significantly increased VGLL4 mRNA levels relative to negative control cells (Fig. 5*E*). Actinomycin D assays demonstrated that the stability of VGLL4 mRNA was significantly enhanced upon FTO loss (Fig. 5*F*). Furthermore, FTO knockdown also elevated VGLL4 protein levels relative to controls (Fig. 5, *G* and *H*). These findings suggest that FTO mediates the regulation of VGLL4 through an m6A-dependent pathway in TNBC.

**Figure 6:**
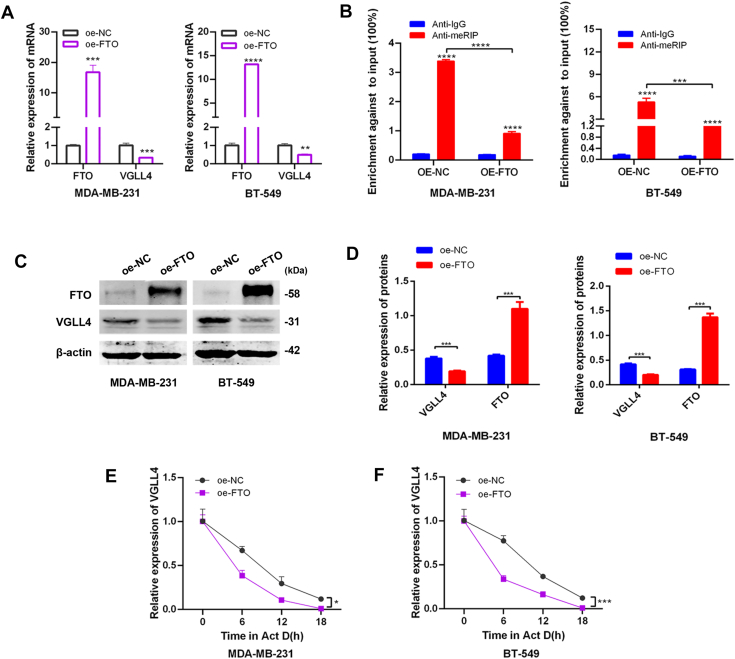
**The effects of FTO overexpression on VGLL4 m6A modification, stability, and expression.***A*, the mRNA levels of VGLL4 and FTO were evaluated in MDA-MB-231 and BT-549 cell lines with FTO overexpression. *B*, the enrichment of VGLL4 m6A levels were evaluated in MDA-MB-231 and BT-549 cell lines with FTO overexpression. *C*–*D*, the protein levels of VGLL4 and FTO were evaluated after FTO overexpression in MDA-MB-231 and BT-549 cells. *E–F*, analysis of VGLL4 mRNA stability by RT-qPCR after Actinomycin D treatment in FTO-overexpressed MDA-MB-231 and BT-549 cells. *∗p < 0.05, ∗∗p < 0.01, ∗∗∗p < 0.001, ∗∗∗∗p < 0.0001.*

Furthermore, FTO overexpression in both MDA-MB-231 and BT-549 cells led to a significant reduction in VGLL4 at multiple levels. Specifically, it downregulated VGLL4 mRNA (Fig. 6*A*), decreased its m6A modification (Fig. 6*B*), and lowered VGLL4 protein expression (Fig. 6, *C* and *D*). Consistent with these findings, Actinomycin D assays confirmed that FTO overexpression markedly reduced the stability of VGLL4 mRNA (Fig. 6, *E* and *F*).

### FTO regulates the VGLL4-STAT3 signaling axis in TNBC cells

VGLL4, a transcriptional cofactor of the VGLL family, has been established as a tumor suppressor in TNBC. Current evidence indicates that the STAT3 signaling pathways play a central role in the tumorigenesis and progression of TNBC (16). We previously showed that VGLL4 suppresses STAT3 signaling in TNBC through a direct interaction with STAT3, inhibiting its phosphorylation and subsequent downstream transcriptional activity (17).

**Figure 7:**
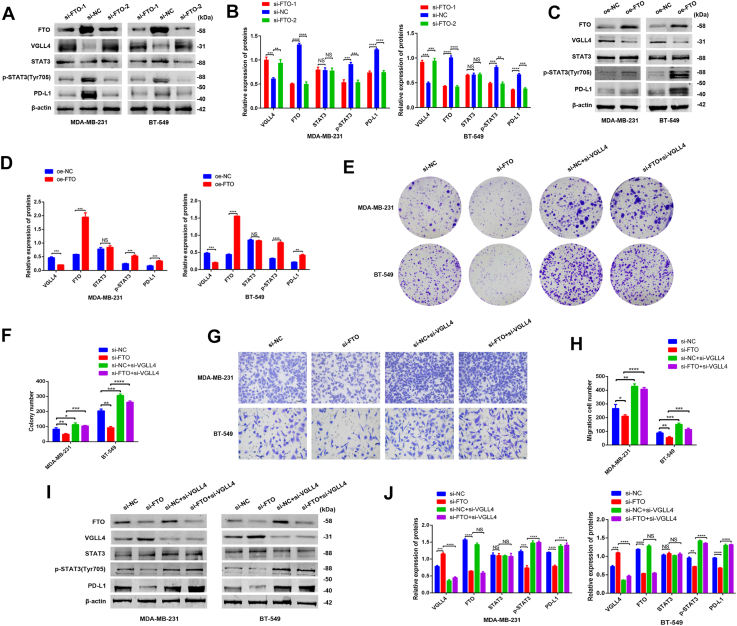
**FTO promotes TNBC progression *via* VGLL4/STAT3/PD-L1 axis.***A–B*, the protein levels of VGLL4, STAT3, p-STAT3 and PD-L1 were evaluated in MDA-MB-231 and BT-549 cell lines after FTO knockdown. *C–D*, the protein levels of VGLL4, STAT3 p-STAT3 and PD-L1 were evaluated in MDA-MB-231 and BT-549 cell lines with FTO over-expression. *E–H*, functional rescue assays: colony formation assay (*E–F*), migration assay (*G–H*). *I–J,* Western blot showed that VGLL4 siRNA can partially rescue the si-FTO-induced upregulation of VGLL4 and downregulation of p-STAT3 and PD-L1 in MDA-MB-231 and BT-549 cell lines. *∗p < 0.05, ∗∗p < 0.01, ∗∗∗p < 0.001, ∗∗∗∗p < 0.0001.* NS, not significant.

To determine whether FTO regulates the VGLL4-STAT3 signaling axis, we measured its activity following FTO knockdown and overexpression in TNBC cells. Western blot analysis revealed that silencing FTO led to a significant increase in VGLL4 protein levels, along with a concurrent decrease in the expression of p-STAT3 and PD-L1 (Fig. 7, *A* and *B*), while FTO overexpression substantially reduced VGLL4 expression and increased the levels of p-STAT3 and PD-L1 (Fig. 7, *C* and *D*). These findings indicated that FTO might regulate STAT3 expression *via* regulating VGLL4 in TNBC cells. Furthermore, using TCGA-TNBC data(https://compbio.cn/timer3/), we found that FTO expression positively correlates with CD8+ T, CD4+ T cell infiltration and expression of immune checkpoint gene CD274 (known as PD-L1) (Fig. S1, *E*–*G*). These data provide preliminary support for FTO's potential immunomodulatory role.

### Silencing VGLL4 rescued the tumor-suppressive phenotype induced by FTO knockdown in TNBC

To validate that FTO promotes TNBC progression *via* the VGLL4/STAT3 axis, we performed rescue experiments by co-transfecting siRNA of FTO and VGLL4 into MDA-MB-231 and BT-549 cells. We found that VGLL4 siRNA partially rescued FTO siRNA-mediated inhibition of TNBC cell proliferation and migration (Fig. 7, *E*–*H*). Similarly, VGLL4 siRNA attenuated the FTO siRNA-induced upregulation of VGLL4 protein expression and downregulation of p-STAT3 levels (Fig. 7, *I* and *J*). In summary, our data suggest that FTO promotes the progression of TNBC, which is associated with VGLL4 downpregulation and STAT3 phosphorylation activation.

## Discussion

Clinically, TNBC exhibits a more aggressive clinical course than other subtypes, characterized by significantly higher rates of recurrence, metastasis, and ultimately, cancer-related death (18). Notably, the 5-year survival rate for non-metastatic TNBC can reach 81%, whereas that for metastatic TNBC falls below 30% (19). The lack of effective treatment strategies hampers the improvement of overall survival in TNBC patients, highlighting the paramount importance of identifying novel therapeutic targets and biomarkers to prevent risk of TNBC.

Dysregulated m6A levels have been linked to multiple malignancies, including breast, lung, and gastric cancers, as well as acute myeloid leukemia (9). The FTO was the first identified m6A demethylase in eukaryotes. Accumulating evidence indicates that FTO is closely associated with tumorigenesis (20), proliferation, and differentiation. Its high expression in certain malignancies, such as acute myeloid leukemia (21), breast cancer (22), and gastric cancer (23), may influence tumor proliferation and differentiation. Our study revealed that FTO expression is significantly upregulated in TNBC tumor tissues compared to adjacent normal tissues, and its expression level is positively correlated with Ki-67 index, lymph node metastasis, and TNM stage. KM plotter online survival analysis demonstrated that high FTO expression is associated with lower DFS and OS in patients with TNBC; however, among other molecular subtypes, there was no significant difference in overall survival between the high- and low-FTO expression groups. Thus, FTO may represent a potential poor-prognosis biomarker in TNBC.

Furthermore, functional experiments demonstrated that cellular proliferation and migration were significantly inhibited after knockdown FTO in TNBC cells. Conversely, overexpression of FTO promoted cell proliferation and migration. *In vivo*, FTO overexpression accelerated the growth of xenograft tumors. We identified that FTO markedly modulates VGLL4 expression by mediating m6A modification. However, whether FTO promotes TNBC metastasis *in vivo* warrants future investigation using orthotopic or tail vein models with FTO-manipulated cells.

The VGLL family consists of four mammalian transcriptional cofactors (VGLL1-4) (24) that are involved in the tumorigenesis and progression of BC. For example, high VGLL1 expression has been associated with shorter survival times for patients with various malignancies, including TNBC and endometrial carcinoma, serving as a marker of adverse prognosis (25). Our previous studies demonstrated that VGLL4 acts as a tumor suppressor in TNBC by interacting with STAT3 and inhibiting STAT3 phosphorylation (17). The evidence indicates that the STAT3 signaling pathways play a central role in the tumorigenesis and progression of TNBC (16). Further experiments showed that knockdown of FTO in TNBC cells led to upregulated expression of both VGLL4 protein and its m6A-modified mRNA, accompanied by decreased phosphorylation of STAT3 and reduced PD-L1 expression. These findings suggest that FTO drives TNBC progression by m6A-mediated oncogenic activation of the VGLL4-STAT3 pathway, promoting PD-L1 expression.

While our data strongly support that VGLL4 is a downstream target of FTO *via* m6A modification, the precise m6A residue(s) within VGLL4 transcript that are directly demethylated by FTO remain to be identified. Site-directed mutagenesis of the predicted m6A motifs (*e.g.*, RRACH sequences) will be required in future studies to establish the direct molecular link at single-nucleotide resolution. It is worth noting that TNBC is molecularly heterogeneous, comprising distinct subtypes (*e.g.*, Lehmann six-subtype or FUSCC four-subtype classifications). While we identified the FTO-VGLL4-STAT3 axis as a key oncogenic pathway in TNBC, we did not systematically compare its significance across these subtypes. The cell lines used (MDA-MB-231 and BT-549) belong to mesenchymal-related subtypes (MSL and M, respectively). Whether this axis plays a more prominent role in basal-like, immunomodulatory, or LAR subtypes remains to be investigated in future studies using subtype-specific models and patient cohort analyses.

The targeting of the PD-1/PD-L1 immune checkpoints, a key mechanism used by tumors to suppress T-cell activity, has emerged as a transformative strategy in cancer treatment, including TNBC. Many studies have reported that constitutive PD-L1 activation, driven by the dysregulation of key oncogenic and tumor-suppressive pathways such as PTEN deletions (26), PI3K/AKT mutations (27), and JAK-STAT3 signaling activation (28), represents a general mechanism in tumorigenesis. VGLL4 could inhibit PD-L1 expression by suppressing STAT3 activation, thereby enhancing the efficacy of anti-PD-1 antibody immunotherapy in TNBC (28). Here, we discovered that FTO activates STAT3 and PD-L1 expression by suppressing VGLL4 expression in an m6A-dependent manner. This indicates that FTO may be a promising therapeutic target for TNBC. In TNBC, the expression level of PD-L1 is usually positively correlated with the number of infiltrating CD8+ T cells (29). CD8+ T cells are important effector cells in anti-tumor immunity, and higher CD8+ T cell infiltration indicates a better immune response and clinical outcome. Consistent with this immune-related landscape, FTO expression also positively correlates with CD8+ T cell, CD4+ T cell infiltration, and the immune checkpoint gene CD274 (known as PD-L1) in TNBC, suggesting a potential immunomodulatory role. However, direct evidence for enhanced immunotherapy efficacy requires immunocompetent models.

Several studies have investigated FTO in BC, revealing context-dependent functions. Consistent with our pro-tumor findings, previous research has reported that FTO promotes TNBC *via* BNIP3 and NFKBIE, respectively (30, 31). Another study also showed that FTO enhances C/EBPβ-LIP translation to perform oncogenic functions in breast cancer cells (32). In contrast, some work has shown that FTO suppresses TNBC through the miR-17-5p/ZBTB4 axis (33), indicating that FTO can exert opposing effects depending on the dominant downstream target. Additionally, it has been demonstrated that the oncometabolite D-2HG inhibits FTO to drive metastasis (34), placing FTO within an upstream metabolic regulatory network. Together, these findings support a model where FTO acts as a context-dependent modulator in TNBC. Our study identifies VGLL4 as a novel m6A target of FTO, linking it to VGLL4/STAT3 signaling and expanding the known oncogenic network of FTO.

In summary, this study systematically reveals the mechanism through which FTO drives TNBC progression *via* m6A-mediated regulation of the VGLL4-STAT3-PD-L1 signaling axis. These findings establish the oncogenic role of FTO in TNBC and provide a rationale for targeting the FTO or this signaling pathway.

## Experimental procedures

### Clinical samples

A total of 51 matched pairs of triple-negative breast cancer (TNBC) specimens and corresponding adjacent normal tissues were collected from patients recruited at the Breast Disease Center of the Affiliated Hospital of Qingdao University (Qingdao, China). Individuals who had received prior neoadjuvant therapy were excluded from the study. All tissue samples were immediately snap-frozen in liquid nitrogen after resection and stored at −196 °C until further processing. This study was conducted in accordance with the ethical principles of the Declaration of Helsinki and was approved by the Institutional Ethics Committees of Affiliated Hospital of Qingdao University (Approval No. QYFY-WZLL-30430). All participants provided written informed consent before enrollment in the study.

### Cell culture and transfection

All BC cell lines (MDA-MB-231, BT-549, HCC-1937, MDA-MB-468, HS578T, MCF7, T47D and SK-BR-3), normal breast epithelial cell line (MCF-10A), and HEK-293T cells were acquired from the Chinese Academy of Sciences . All the cells were cultured in recommended media supplemented with 10% Fetal Bovine Serum (FBS; Gibco) and 1% penicillin-streptomycin (PS; Sigma) in a 5% CO2 incubator at 37 °C. BT-549 and MDA-MB-231 were selected as complementary models for FTO manipulation based on their differential expression, high transduction efficiency, and subtype diversity. Two independent small interfering RNA targeting FTO (si-FTO-1 and si-FTO-2) and the negative control (si-NC) were purchased from IBSbio . The lentiviral vector based overexpressing FTO (FTO), shRNAs targeting FTO (shFTO) and an empty vector used as a negative control (NC) were designed from ZORIN Biotech. VGLL4 knockdown was performed using siRNA (Sangon Biotech). Lipofectamine 2000 (Invitrogen) was used for transfection, according to the manufacturer’s instructions. Lentiviral particles were produced in HEK-293T cells by co-transfecting the lentiviral construct and the packaging kits according to the manufacturer’s instructions.

### Reverse transcription-Quantitative Real-Time Polymerase Chain Reaction (RT-qPCR)

Total RNA was extracted from tissues and cells *via* TRIzol reagent (Invitrogen, USA). cDNA was generated through reverse transcription of the extracted RNA employing the Hifair III first Strand cDNA Synthesis SuperMix (Yeasen, China). Gene expression was quantified by qPCR using Hieff qPCR SYBR Green Master Mix (Yeasen, China). The 2^−ΔΔCt^ calculation method was applied for data analysis, with normalization to the internal reference genes β-actin (for mRNAs).

### Actinomycin D assay

To assess transcript stability, MDA-MB-231 and BT-549 cells were exposed to 2 μg/ml actinomycin D (Merck, Germany) to inhibit transcription. RNA was isolated at 0, 4, 8, and 12 h post-treatment, and its relative levels were quantified by RT-qPCR.

### MTT assay

TNBC cells were plated in 96-well plates at 2000 cells per well in 200 μl of medium. At 0, 24, 48, 72, and 96 h post-seeding, 20 μl of MTT reagent (YEASEN, China) was added to each well and incubated for 4 to 6 h at 37 °C. Thereafter, the supernatant was carefully aspirated and replaced with 150 μl of DMSO (Sangon) to solubilize the formazan crystals. The optical density (OD) at 490 nm was then recorded using a microplate spectrophotometer (BioTek).

### Colony formation assay

TNBC cells were seeded into 6-well plates at 1000 cells per well. Following a culture period of ∼10 days, the colonies were subsequently washed with phosphate-buffered saline (PBS), fixed in 95% ethanol, and stained using a 0.1% crystal violet solution. Representative wells were photographed, and the number of visible colonies was counted.

### Wound healing assay

A wound healing assay was conducted using TNBC cells cultured in 6-well plates. Upon reaching approximately 95% confluency, a uniform scratch was generated in the cell monolayer using a 200 μl pipette tip. The cells were subsequently maintained in DMEM containing 2% FBS. Wound closure was monitored and evaluated at the same position under a microscope at 0, 12, 24, 36, and 48 h post-wounding.

### Transwell assay

MDA-MB-231 and HCC-1937 cells were seeded into the upper chamber in 200 μl of serum-free medium, while the lower chamber was filled with medium supplemented with 10% FBS. Following incubation for 12 to 16 h, cells that had migrated to the lower surface of the membrane were fixed with 4% paraformaldehyde (PFA; Beyotime, China), stained with 0.1% crystal violet (Beyotime, China), and imaged under a microscope (Leica Microsystems) at 200× magnification. The number of migrated cells was quantified by counting five randomly selected fields per well.

### Methylated RNA immunoprecipitation (MeRIP)-RT-qPCR

The MeRIP assay was performed using an riboMeRIP™ m6A Transcriptome Profiling Kit (RiboBio, China) according to the manufacturer’s instructions. Briefly, fragmented RNAs were incubated with magnetic beads coated with 5 μg control IgG antibody and anti-m6A antibody with rotation at 4 °C for 2 to 3 h. Next, the RNAs were eluted, and the enrichment of m6A-modified VGLL4 mRNA was quantified by RT-qPCR. Data were quantified using the 2^−ΔΔCt^ method. Calculate % (IP/Input) = 2^(Ct(Input) - Ct(IP))^ × Dilution Factor × 100%.

### Western blot

Total protein was extracted with RIPA lysis buffer (Beyotime) containing PMSF (Beyotime). The proteins were separated on 10% SDS-PAGE gels and subsequently transferred onto nitrocellulose membranes (Beyotime). The membranes were blocked with 5% non-fat milk for 1 h at room temperature, followed by an overnight incubation at 4 °C with primary antibodies, including: anti-VGLL4 (1:1000, Abclonal), anti-STAT3 (1:1000, Proteintech), anti-phospho-STAT3(Tyr705) (1:1000, Proteintech, USA), anti-PD-L1 (1:1500, Proteintech), and anti-β-actin (1:10,000, Abclonal). After washing, the membranes were incubated with the corresponding secondary antibodies for 1 h at room temperature. Protein bands were visualized and analyzed using an Odyssey Infrared Imaging System (LI-COR Biosciences).

### Immunohistochemistry (IHC)

Immunohistochemical staining was conducted on formalin-fixed, paraffin-embedded tissues. Briefly, sections were deparaffinized, rehydrated, and treated with hydrogen peroxide to block endogenous peroxidase. After PBS washing, the slides were blocked with goat serum and incubated with primary antibodies at 4 °C overnight. Subsequently, sections were incubated with biotinylated secondary antibodies and streptavidin–HRP complex using a Biotin-Streptavidin Detection Kit (SP9000, ZSGB-BIO). Detection was performed with a DAB substrate kit (Dako), and five random fields per slide were examined microscopically for evaluation.

### Animal experiments

Four-week-old female BALB/c nude mice were obtained from Shanghai SLAC Laboratory Animal Company (Shanghai, China). Mice were randomly allocated into three groups and housed in individually ventilated cages under specific pathogen-free conditions, with a controlled environment (22–23 °C, 50% humidity, 12 h:12 h light-dark cycle) and free access to food and water. All animal experiments were performed in accordance with the guidelines for the welfare and use of animals in cancer research. All experimental procedures were approved by the Animal Ethics Committee of the Afffliated Hospital of Qingdao University (Approval No. AHQU20221101SHM). MDA-MB-231 cells (1 × 10^6^) with stable FTO overexpression or knockdown, along with their negative controls, were injected into the second mammary fat pad of mice. Mice health and behavioral status were monitored twice daily throughout the study. Tumor volume was measured three times weekly and calculated using the formula: volume (mm^3^) = (width^2^ × length)/2. The predefined humane endpoint in our study was a tumor diameter >15 mm. At 5 weeks post-inoculation, all mice were anesthetized with isoflurane (5% for induction, 2.5% for maintenance) and euthanized by cervical dislocation. No spontaneous mortality occurred prior to the scheduled endpoint. Death was confirmed by the absence of vital signs (including cessation of both heartbeat and respiration for >1 min). No adverse events occurred during the experiment.

### Bioinformatics analysis

The pan-cancer prognostic association of FTO was evaluated using TCGA data from the TISCH database (http://tisch.comp-genomics.org). The association between FTO expression and overall survival (OS) across breast cancer molecular subtypes was analyzed *via* the Kaplan-Meier Plotter (https://www.kmplot.com/analysis/). Genetic correlates of FTO in TNBC were profiled using cBioPortal database (http://www.cbioportal.org/). In addition, high-throughput sequencing expression profiles of the m6A demethylase FTO were retrieved from the GEO database (http://www.ncbi.nlm.nih.gov/geo/). The m6A modification level of VGLL4 was predicted using the SRAMP database (http://www.cuilab.cn/sramp/).

### Statistical analysis

Data from a minimum of three independent experiments were processed with GraphPad Prism (version 8.3.0, USA). Results are expressed as mean ± standard deviation (SD), and statistical significance was defined as *p* < 0.05. The relationship between FTO expression and clinicopathological features was assessed using the Chi-square test or Fisher’s exact test, as appropriate. Comparisons within paired samples were performed using the Wilcoxon matched-pairs signed rank test, while unpaired groups were compared using the unpaired Student’s *t* test.

## Data availability

The data used to support the findings of this study are included in the article and its supplementary files.

## Supporting information

This article contains supporting information.

## Conflict of interest

The authors declare that they have no conflicts of interest with the contents of this article.
