# Supplementary material for: FTO promotes cancer progression by regulating VGLL4 m6A levels to activate STAT3 signaling in triple-negative breast cancer
Source: J Biol Chem. 2026 Jun 9;302(8):113242. doi: 10.1016/j.jbc.2026.113242 (PMC13355748; doi:10.1016/j.jbc.2026.113242)
Supplement: Supplementary Figure S1 [file mmc1.doc]

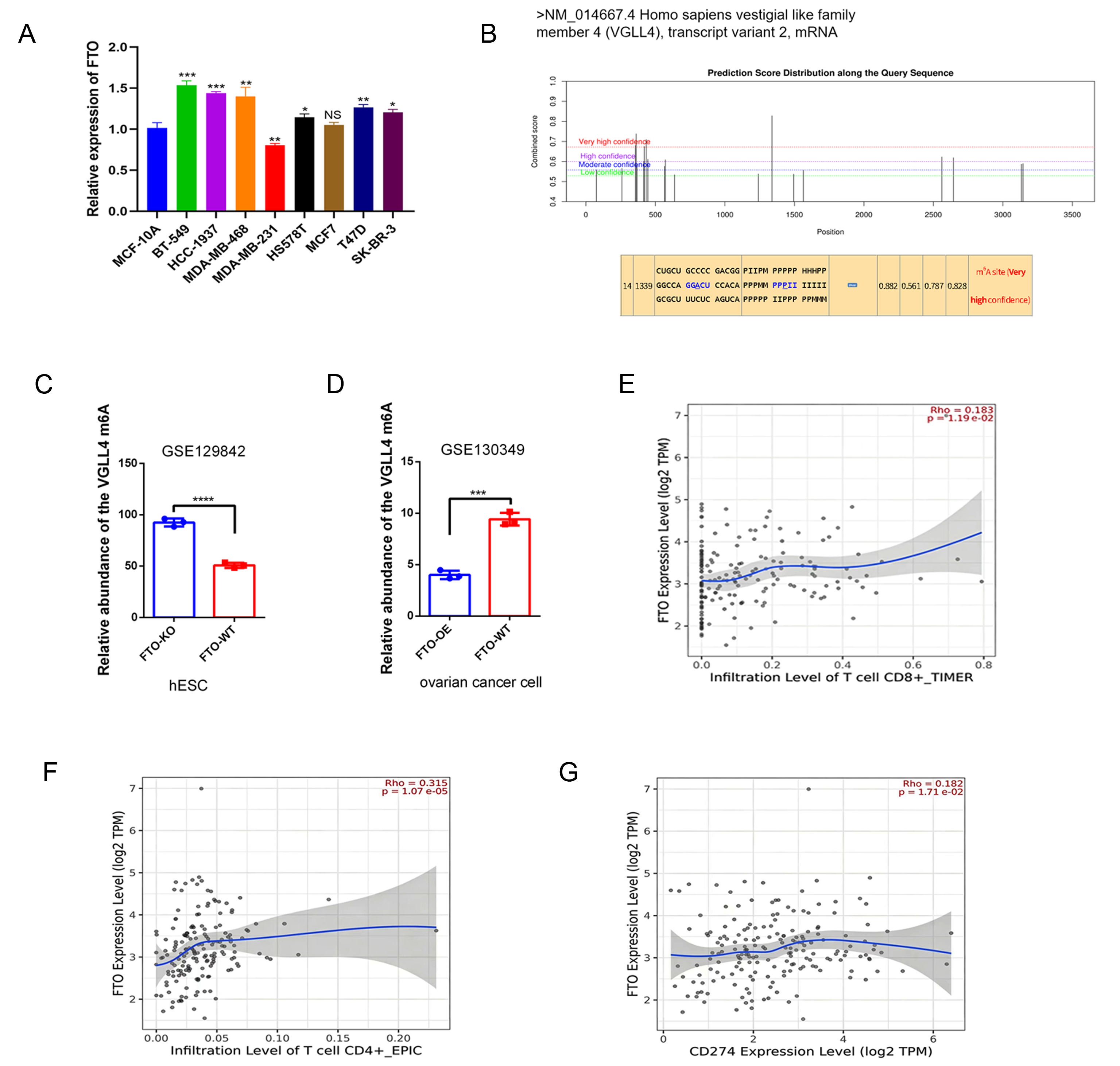


**Fig. S1** *A*, relative expression levels of FTO in breast cancer cell lines. *B*, **SRAMP databases showed that VGLL4 is enriched in m6A modification sites.** *C*, **GSE129842 dataset revealed a marked increase in VGLL4 m6A modification levels in FTO-knockout (KO) groups compared to wild-type (WT) controls. *D*, GSE130349 dataset demonstrated a significant decrease in VGLL4 m6A modification abundance following FTO overexpression (OE) relative to WT groups. *E-F,* FTO expression positively correlates with CD8+ T cell (E, r = 0.183, p < 0.05) and CD4+ T cell infiltration (F, r = 0.315, p < 0.0001). *G,*FTO expression shows a positive correlation with immune checkpoint gene CD274 expression (r = 0.182, p < 0.05). Correlation coefficients were calculated using Spearman/Pearson correlation analysis.****p < 0.05,**p < 0.01, *** p < 0.001, **** p < 0.0001.*NS: not significant.
