# Supplementary material for: FTO promotes cancer progression by regulating VGLL4 m6A levels to activate STAT3 signaling in triple-negative breast cancer
Source: J Biol Chem. 2026 Jun 9;302(8):113242. doi: 10.1016/j.jbc.2026.113242 (PMC13355748; doi:10.1016/j.jbc.2026.113242)
Supplement: Supplementary Table S1 [file mmc2.pdf]

Table S1 Primers, siRNAs and all negative controls used in this study

Name		Sequences
β-actin	Primer-Forward	5’-CAGAGCCTCGCCTTTGCC-3’
	Primer- Reverse	5’-GTCGCCCACATAGGAATC-3’
VGLL4	Primer-Forward	5'-AACTGCAACCTCTCGCACTG-3'
	Primer- Reverse	5'-GCTCGGGCTCCTTGTAATTCT-3'
VGLL4-m6A	Primer-Forward	5'- CTCTGATGCAGGTCTGCTG -3'
	Primer- Reverse	5'- CATCATGGCTTGTGACTGAGA-3'
FTO	Primer-Forward	5'- GGATGAGCCAGCTTCACTGT-3'
	Primer- Reverse	5'- AGAAGGGTGCGATTTCTGGG-3'
si-RNA NC	Sense	5’-UUCUCCGAACGUGUCACGUTT-3'
	Anti-sense	5’-ACGUGACACGUUCGGAGAATT-3'
si-FTO- 1	Sense	5’-CCAAAGAUGAUGAAUUCUATT-3’
	Anti-sense	5’-UAGAAUUCAUCAUCUUUGGTT-3’
si-FTO- 2	Sense	5’-AGCUGAAAUAUCCUAAACUTT-3'
	Anti-sense	5’-AGUUUAGGAUAUUUCAGCUTT-3'
si-VGLL4	Sense	5’ -CCACGUCUCCAAAAUGAGUTT- 3’
	Anti-sense	5’ -ACUCAUUUUGGAGACGUGGTT- 3’
si-RNA Negative control	Sense	5’ -UUCUCCGAACGUGUCACGUTT-3’
	Anti-sense	5’-ACGUGACACGUUCGGAGAATT-3’
